# LGALS3BP/90K suppresses porcine reproductive and respiratory syndrome virus replication by enhancing GP3 degradation and stimulating innate immunity

**DOI:** 10.1186/s13567-025-01556-2

**Published:** 2025-06-20

**Authors:** Qingyu Fan, Haiyan Wang, Hanze Liu, Jinzhi Li, Juan Huang, Ying Yu, Zhi Cao, Qiaoya Zhang

**Affiliations:** 1 College of Veterinary Medicine, Qingdao Agricultural University, Qingdao, 266109 China; 2 China Animal Health and Epidemiology Center, Qingdao, 266032 China

**Keywords:** PRRSV, LGALS3BP/90K, viral replication, proteasomal degradation, innate immunity

## Abstract

Porcine reproductive and respiratory syndrome virus (PRRSV) seriously affects the world pig industry, and existing vaccines are not enough to protect against PRRSV efficiently. Identifying host factors that inhibit PRRSV infection may provide potential targets for antiviral therapies. Galectin 3 binding protein (LGALS3BP), also referred to as 90K, is a multifunctional protein that plays significant roles in viral infections, but its role in PRRSV infection is not clearly defined. In this study, we found that 90K expression increased during PRRSV infection. Additionally, 90K inhibits PRRSV replication by restricting both viral RNA synthesis and viral assembly. Further studies showed that 90K can interact with various PRRSV structural proteins, such as GP2, GP3, and GP5. Moreover, 90K targets K217 in GP3 for K48-linked ubiquitination, facilitating GP3 degradation through the proteasomal pathway. Meanwhile, we found that 90K induces the expression of type I interferon (IFN) and inflammatory cytokines by activating NF-κB and IRF3. Overall, our findings suggest that 90K plays a negative role in PRRSV replication by inducing PRRSV-GP3 degradation and activating innate immune responses.

**Supplementary Information:**

The online version contains supplementary material available at 10.1186/s13567-025-01556-2.

## Introduction

The porcine reproductive and respiratory syndrome virus (PRRSV), responsible for PRRS, was first identified in the 1980s. This virus has consistently threatened the global pig industry for nearly 40 years, resulting in significant annual economic losses [2, 27]. According to the 10th classification report of the International Committee on Taxonomy of Viruses (ICTV), PRRSV is classified under the genus *Betaarterivirus*, which includes two distinct species: *Betaarterivirus suid 1* (PRRSV-1) and *Betaarterivirus suid 2* (PRRSV-2) [18]. The genome of PRRSV is a single-stranded, positive-sense RNA, approximately 15 kb in length. It has at least 11 open reading frames (ORFs), encoding at least 16 nonstructural proteins (nsps), as well as 8 structural proteins [8, 9]. The major envelope proteins, GP5 and M, can form a disulfide-linked heterodimer, playing a crucial role in the assembly of PRRSV [5, 29]. Minor envelope proteins, including GP2a, GP3, and GP4, form a heterotrimer on the virion envelope [34, 38].

Currently, vaccination remains the primary strategy to control PRRS. Many commercial vaccines are available, but they do not provide effective or lasting protection. There is an urgent need to develop effective and safe methods to control PRRSV. Identifying host factors that inhibit PRRSV infection may provide potential targets for antiviral therapies. Numerous host proteins are known to inhibit PRRSV infection through various mechanisms. For example, the proteasome subunit beta type 4 (PSMB4) restricts PRRSV replication by degrading PRRSV-nsp1α and activating type I interferon (IFN) signaling pathways [36]. Non-POU domain-containing octamer-binding protein (NONO) enhances IRF3-mediated IFN-β activation by interacting with PRRSV-N to inhibit PRRSV infection [16]. Programmed cell death 4 (PDCD4) counteracts PRRSV replication by interacting with eukaryotic translation initiation factor 4A (eIF4A) and disrupting the translation initiation of the PRRSV genome [32]. Cholesterol 25-hydroxylase (CH25H) inhibits PRRSV infection by producing 25HC and promoting the degradation of PRRSV-nsp1α through the proteasome pathway [17].

LGALS3BP/90K is a multifunctional glycoprotein originally identified in cancer progression [13]. Notably, 90K expression has also been found to increase during several viral infections, including human immunodeficiency virus (HIV), dengue virus (DENV), influenza A virus (IAV), hantavirus and severe acute respiratory syndrome coronavirus 2 (SARS-Cov-2) [3, 14, 20, 24, 25, 35]. Studies have also examined the roles of 90K in viral infections. For example, 90K disrupts the processing of gp160/Env and decreases the incorporation of Gag into virions. This interference impacts both the release of new virions and their ability to infect cells in HIV infection. [21, 31]. 90K can also increase survival of mice infected with IAV via antiviral innate immune signaling [21]. In the case of SARS-CoV-2, however, 90K shows no detectable antiviral properties in the context of SARS-CoV-2 infection, suggesting that 90K may not always play an antiviral role during viral infections [3]. Overall, studies on the mechanisms by which 90K inhibits viral infection are rare, and the role of 90K during PRRSV replication remains unknown.

In this study, we showed that PRRSV infection induced 90K expression, which in turn limited PRRSV replication. We found 90K could interact with PRRSV structural proteins and enhance GP3 degradation. Moreover, 90K could upregulate cytokine expression by activating the NF-κB and IRF3 signaling pathways. In summary, our study elucidates the antiviral mechanism of this protein in controlling PRRSV replication and suggests that 90K may serve as a promising new antiviral target.

## Materials and methods

### Cells and viruses

Primary porcine alveolar macrophages (PAMs) were maintained in RPMI 1640 medium (HyClone, USA) containing 10% fetal bovine serum (Vazyme, China) and penicillin–streptomycin [37]. The 3D4/21-CD163 cells are a type of immortalized PAMs. They consistently express the CD163 receptor and can be infected by PRRSV. Additionally, these cells use the same culture medium as primary PAMs. MARC-145 and HEK-293T cells were maintained in Dulbecco’s modified Eagle’s medium (HyClone, USA) with the same supplements as PAMs, at a temperature of 37 °C and 5% CO_2_. The strains of PRRSV-2, including BB0907 (highly pathogenic, GenBank: HQ315835.1), S1 (classical, GenBank: DQ459471.1), and FJ1402 (NADC30-like, GenBank: KX169191.1), were provided by Prof. Ping Jiang from Nanjing Agricultural University. Amplification and titration of the PRRSV were conducted with MARC-145 cells. Unless otherwise specified, PRRSV refers to the BB0907 strain in this study.

### Plasmids, siRNA and transfection

The cDNA of PAMs was used to amplify the porcine 90K encoding sequence, which was then cloned into the pcDNA3.1(+) vector to create the HA-tagged plasmid named 90K-HA. Flag-tagged PRRSV structural protein expression plasmids were developed and preserved in our laboratory. Expression plasmids encoding mutant GP3 with alanine substitutions were achieved by PCR using PrimeSTAR® HS DNA Polymerase from Takara, Japan. Primers used for plasmid construction are presented in Additional file 1. The Myc-tagged ubiquitin (Ub) expressing plasmid, pCMV-Myc-Ub, and its mutants, pCMV-Myc-Ub (K48) and pCMV-Myc-Ub (K63), were purchased from HonorGene (Changsha, China).

Knockdown of endogenous 90K in MARC-145 cells was achieved through transfection with 90K-targeting siRNA (si90K), while non-targeting siRNA (siNC) was employed as the negative control. The siRNAs were synthesized by Sangon (Shanghai, China). Additional file 2 contains the siRNA sequences. Using RNATransMate from Sangon (Shanghai, China), 50 pmol of siRNA or siNC was used to transfect MARC-145 cells in 24-well plates.

### Quantitative real‐time PCR (qRT‒PCR)

Total RNA was isolated using a Bioer RNA extraction kit (Hangzhou, China), and then employed for cDNA synthesis with the HiScript II 1st Strand cDNA Synthesis Kit from Vazyme (Nanjing, China). According to the manufacturer’s guidelines, the AceQ qPCR SYBR Green Master Mix from Vazyme was utilized for qRT-PCR. The 2^−ΔΔCt^ method was employed to calculate relative gene expression levels, which are displayed as fold changes normalized to β-actin and compared to the control group. The primer sequences used in the qRT‒PCR assays are provided in Additional file 3.

### Reagents and antibodies

The proteasome inhibitor MG132 (HY-13259) and the lysosome inhibitor chloroquine (CQ) (HY-17589A) were sourced from MedChemExpress (New Jersey, USA). The following antibodies were purchased from Abmart (Shanghai, China): HA-tagged mouse monoclonal antibody (mAb) (M20003S), Flag-tagged rabbit polyclonal antibody (R20008S), and antibodies against various signaling molecules such as IκBα (T55026S), p-IκBα (TP56280S), p65 (T55034S), and p-p65 (TP56372S). Antibodies targeting IRF3 (4302 T), TBK1 (3504 T) and p-TBK1 (5483 T) were obtained from Cell Signaling Technology (Danvers, Massachusetts, USA). Goat anti-mouse HRP-conjugated antibody (A0216) and goat anti-rabbit HRP-conjugated antibody (A0208) were sourced from Beyotime Biotechnology (Shanghai, China). Beta-actin rabbit mAb (High Dilution) (AC026) and p-IRF3 rabbit mAb (AP0995) were obtained from ABclonal (Wuhan, China). The following antibodies were purchased from Proteintech (Chicago, USA): LGALS3BP polyclonal antibody (10281-1-AP), CoraLite594-conjugated goat anti-mouse IgG (SA00013-3), and CoraLite488-conjugated goat anti-rabbit IgG (SA00013-2). The mAb that targets double-stranded RNA (dsRNA), known as J2, was procured from Scicons (Sizraku, Hungary). PRRSV-N mAb was kindly provided by Prof. Ping Jiang at Nanjing Agricultural University.

### Western blot (WB) and co-immunoprecipitation (Co‐IP)

Cells cultured in plates were washed three times with cold PBS and then lysed on ice for 30 min (min) using NP-40 with 1 mM PMSF (Beyotime, Shanghai, China). Cell lysates were then collected and centrifuged at 12 000 rpm for 5 min under 4 °C to collect supernatants. The supernatants were loaded onto 12.5% SDS-PAGE gels (Epizyme, Shanghai, China). After electrophoresis, the proteins were transferred to PVDF membranes (Millipore, Boston, USA). After blocking in skimmed milk for 2 h (h), the PVDF membranes were incubated with primary antibodies at 4 °C for the night. After being washed with TBST (1×) from Solarbio (Beijing, China), the membranes were exposed to secondary antibodies for 45 min at room temperature. Following the incubation with secondary antibodies, ECL detection reagents (Tanon, Shanghai, China) were used to visualize the protein bands. For Co-IP, the supernatants were incubated with the corresponding primary antibodies at 4 °C for 4 h, then precipitated overnight at 4 °C using Protein A+G Agarose (Beyotime, Shanghai, China). The agarose beads were gathered through centrifugation, carefully rinsed three times with NP-40 buffer to eliminate unbound proteins, and subsequently utilized for WB analysis.

### Indirect immunofluorescence assay (IFA)

Cells on coverslips were rinsed with PBS and subsequently fixed in 4% paraformaldehyde for 20 min. Following three PBS washes, cells were permeabilized using Immunostaining Permeabilization Solution (Beyotime, Shanghai, China) for 30 min. The cells were exposed to primary antibodies overnight at 4 °C. After rinsing with PBS, they were tagged with fluorescent secondary antibodies (Proteintech, Chicago, USA) for 1 h at 37 °C, and then stained with DAPI (Beyotime, Shanghai, China). Fluorescence images were observed using confocal microscope (Zeiss, Oberkohen, Germany).

### Statistical analysis

All experiments were repeated three times with three biological replicates. The statistical analyses were carried out using GraphPad Prism 8.0 software (San Diego, CA), with results expressed as the mean ± standard deviation (SD). One-way ANOVA or a t-test was used to evaluate statistical significance. *P* < 0.05 (*), *p* < 0.01 (**), and *p* < 0.001 (***) were considered statistically significant.

## Results

### PRRSV infection promotes the expression of 90K

**Figure 1:**
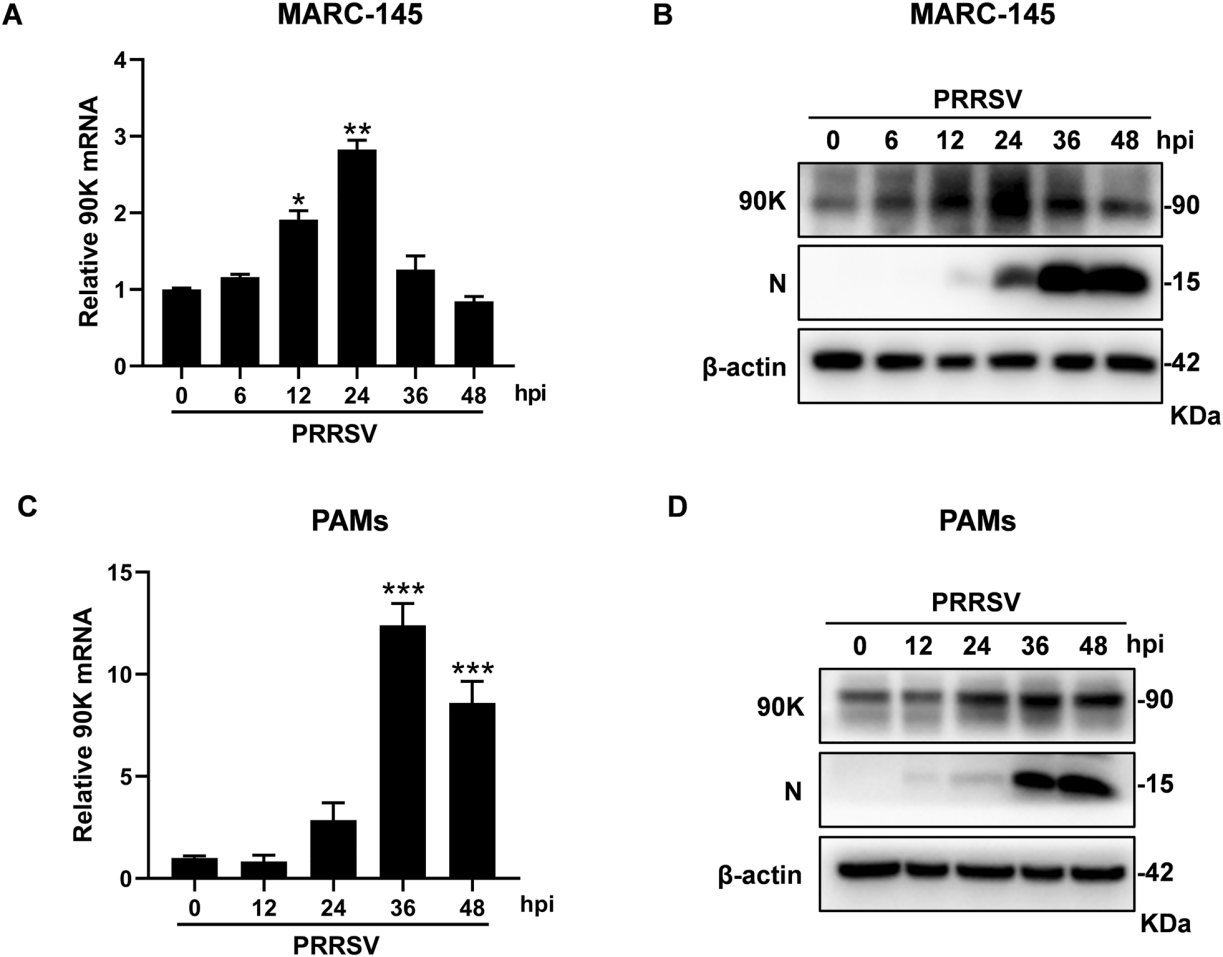
**The expression of 90K can be induced in cells infected with PRRSV.** MARC-145 cells or PAMs were infected with PRRSV BB0907 (MOI = 0.1) for indicated hours. **A** and **C** RNA was extracted from cells and 90K mRNA levels were measured by qRT-PCR. These values were normalized to the levels of β-actin mRNA. The bars represent the means ± SD from three independent experiments. (*, *p* < 0.05; **, *p* < 0.01; ***, *p* < 0.001). **B** and **D** Protein expression levels of endogenous 90K and PRRSV-N were detected by western blot (WB). The antibodies used are listed in the adjacent column. β-actin was used as the internal loading control.

Previous studies suggest that the 90K expression is upregulated in response to various viral infections. Thus, we initially evaluated if PRRSV triggers the expression of 90K. MARC-145 cells were infected with PRRSV BB0907 for indicated hours, and cells were then processed to measure the 90K expression levels. As shown in Figure 1A, the mRNA levels of 90K were significantly upregulated at 12 h post-infection (hpi) and peaked at 24 hpi. Similarly, the protein levels of 90K were upregulated and peaked at 24 hpi, consistent with the mRNA results (Figure 1B). PRRSV-N expression was first detected at 12 hpi and reached its highest level at 48 hpi. To confirm these findings, we investigated the expression of 90K in primary PAMs after infection. As illustrated in Figures 1C and D, both mRNA and protein levels of 90K increased following PRRSV infection in PAMs. These results suggest that PRRSV infection increases 90K expression regardless of cell type.

### 90K overexpression restricts PRRSV replication

**Figure 2:**
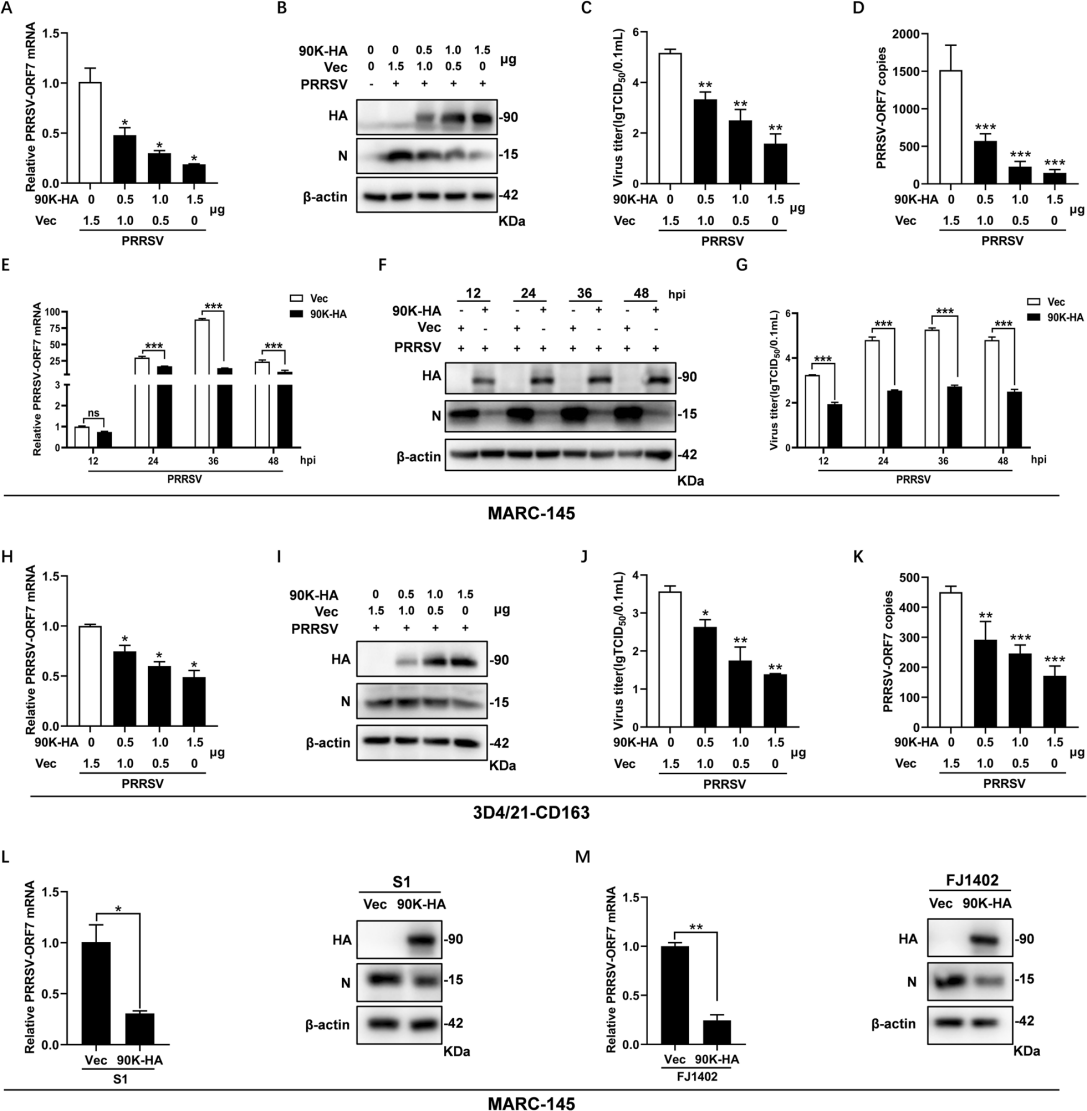
**Overexpression of 90K inhibits PRRSV replication.** **A**–**D** MARC-145 cells were transfected with different doses of HA-tagged 90K expressing plasmid (90K-HA) (0, 0.5, 1, or 1.5 μg), along with an empty vector (Vec) adjusted accordingly. At 24 h post-transfection (hpt), the cells were infected with PRRSV BB0907 (MOI = 1.0). **A** Cells were collected at 24 hpi. The mRNA levels of PRRSV-ORF7 were detected by qRT-PCR. **B** Protein expression levels of 90K-HA and PRRSV-N were detected by WB. **C** Supernatants were harvested for determining viral titers by TCID_50_ assay. **D** PRRSV-ORF7 copies in the supernatant were also determined by qRT-PCR. **E**–**G** MARC-145 cells were transfected with 1 µg of either 90K-HA or Vec. At 24 hpt, cells were infected with PRRSV BB0907 (MOI = 1.0) for indicated hours. Cells and supernatants were collected to access PRRSV replication levels by qRT-PCR (**E**), WB (**F**), and TCID_50_ assay (**G**). **H**–**K** Parallel dose–response experiments were conducted using 3D4/21-CD163 cells. PRRSV-N expression levels were detected by qRT-PCR (**H**) and WB (**I**). Viral titers and PRRSV-ORF7 copies in the supernatant were determined by TCID_50_ assay (**J**) and qRT-PCR (**K**). **L** and **M** MARC-145 cells were transfected with either 90K-HA or Vec and subsequently infected with PRRSV strains S1 or FJ1402 (MOI = 1.0). At 24 hpi, cells were harvested for qRT-PCR and WB analysis. The bars represent the means ± SD from three independent experiments. (*, *p* < 0.05; **, *p* < 0.01, ***, *p* < 0.001).

The discovery that PRRSV induces 90K led us to examine how 90K affects PRRSV replication. MARC-145 cells were transfected with different doses of the 90K expression plasmid (90K-HA) or the empty vector (Vec). Afterwards, the cells were infected with PRRSV BB0907. The qRT-PCR and WB analyses revealed that overexpressing 90K significantly reduced both mRNA and protein levels of PRRSV-N in a dose-dependent manner (Figures 2A and B). Additionally, it effectively decreased the viral titers and the copy number of PRRSV-ORF7 present in the cell supernatant (Figures 2C and D). We then assessed virus replication in MARC-145 cells transfected with a specific dose of 90K-HA at various time points after PRRSV infection. The results indicated that upregulating 90K significantly reduced both PRRSV-N expression levels and virus titers at various time points (Figures 2E–G). The inhibitory effect of 90K overexpression on PRRSV replication was also confirmed in the 3D4/21-CD163 cell line. Similar to the results observed in the MARC-145 cells, 90K overexpression decreased the mRNA and protein levels of PRRSV-N, as well as the viral titers and PRRSV-ORF7 copies in the cell supernatant (Figures 2H–K). We also found that the overexpression of 90K restricted the replication of two other sub-genotype PRRSV strains, S1 (a classical strain) and FJ1402 (an NADC30-like strain) (Figures 2L and M). All these results suggest that 90K plays a negative role in PRRSV replication.

### 90K knockdown facilitates PRRSV replication

**Figure 3:**
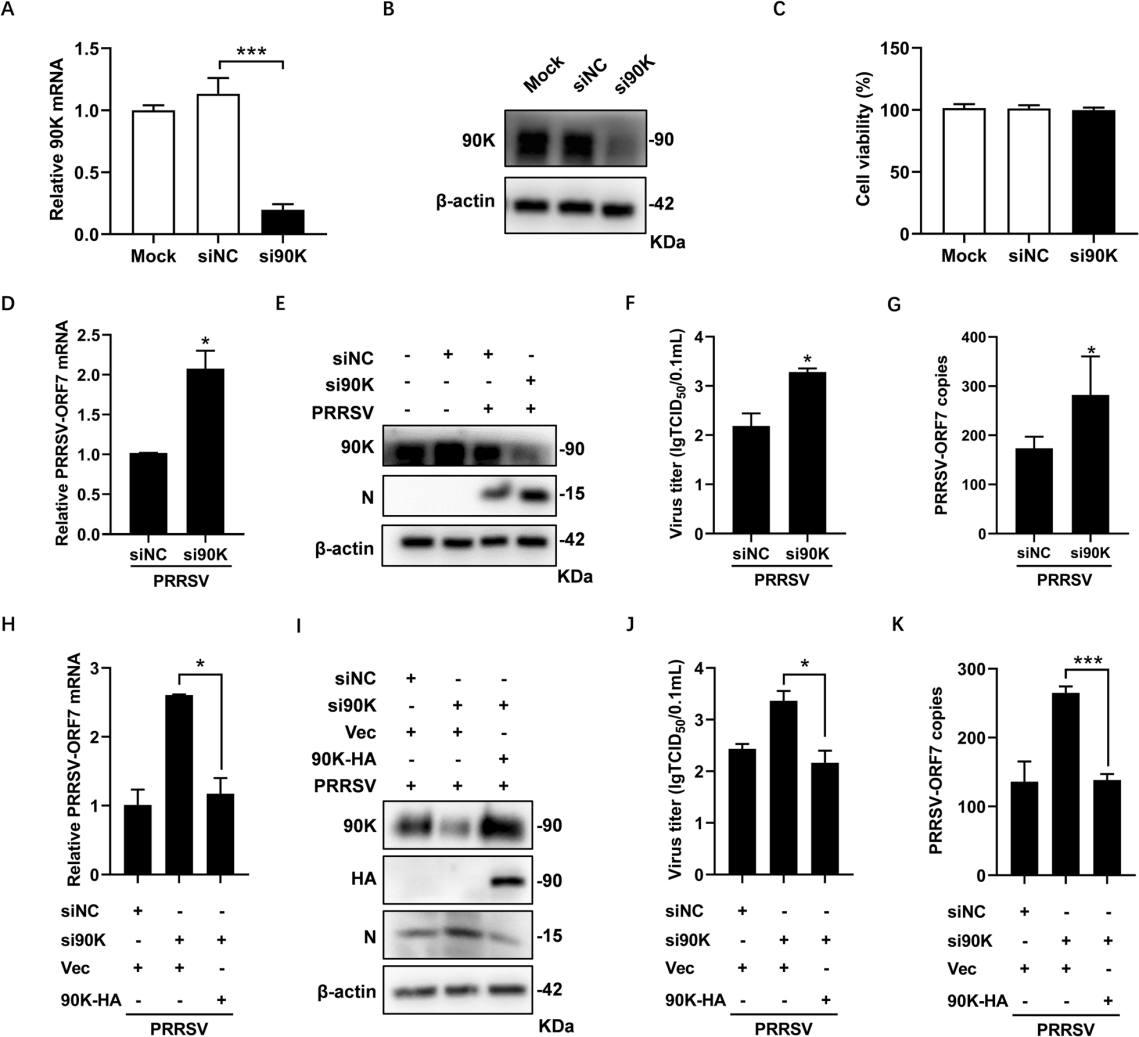
**Knockdown of 90K promotes PRRSV replication.** **A**–**C** MARC-145 cells were transfected with 90K-specific siRNA (si90K) or control siRNA (siNC). Cells were collected after 36 h (h) to access endogenous 90K expression using qRT-PCR (**A**) and WB (**B**). **C** The cytotoxicity of siRNAs on MARC-145 cells was assessed using a Cell Counting Kit-8 (CCK-8). **D**–**G** MARC-145 cells were transfected with either si90K or siNC. After 36 h, the cells were infected with PRRSV BB0907 (MOI = 0.1) for 24 h. Levels of PRRSV-ORF7 mRNA and PRRSV-N protein were measured by qRT-PCR (**D**) and WB (**E**), while viral titers and PRRSV-ORF7 copies in the supernatant were assessed using the TCID_50_ assay (**F**) and qRT-PCR (**G**). **H**–**K** MARC-145 cells were transfected with either si90K or siNC. After 24 h, the cells were transfected with either 90K-HA or Vec for 24 h, and then infected with PRRSV BB0907 (MOI = 0.1) for another 24 h. Levels of PRRSV-ORF7 mRNA and PRRSV-N protein were measured by qRT-PCR (**H**) and WB (**I**), while viral titers and viral RNA in the supernatant were assessed using the TCID_50_ assay (**J**) and qRT-PCR (**K**). The bars represent means ± SD from three independent experiments. (*, *p* < 0.05; **, *p* < 0.01; ***, *p* < 0.001).

To confirm that 90K negatively impacts PRRSV replication, we designed a small interfering RNA targeting the 90K gene (si90K) in MARC-145 cells and assessed its knockdown effect using qRT-PCR and WB analysis. The results indicated that the expression levels of 90K were significantly lower in cells transfected with si90K, suggesting that si90K effectively achieved a high knockdown efficiency (Figures 3A and B). A cell counting kit-8 (CCK-8) was used to measure the potential influence of si90K on cell viability and no cytotoxicity was found (Figure 3C). MARC-145 cells were then transfected with si90K and inoculated with PRRSV. Viral replication was evaluated with qRT-PCR, WB and TCID_50_ assays. According to Figure 3D and E, knocking down 90K increased the mRNA and protein levels of PRRSV-N. Additionally, this knockdown led to higher viral titers and more copies of PRRSV-ORF7 in the cell supernatant (Figures 3F and G). A rescue experiment was conducted to confirm the specificity of the siRNA-mediated knockdown of 90K by re-expressing it in si90K-treated cells. The results demonstrated that re-expressing 90K significantly reversed the enhancement of PRRSV replication. This confirms that the observed effects were specifically due to the knockdown of 90K, rather than off-target effects of the siRNA (Figures 3H–K). Therefore, we conclude that 90K serves as a host restriction factor for PRRSV.

### 90K restricts PRRSV RNA replication and viral assembly

**Figure 4:**
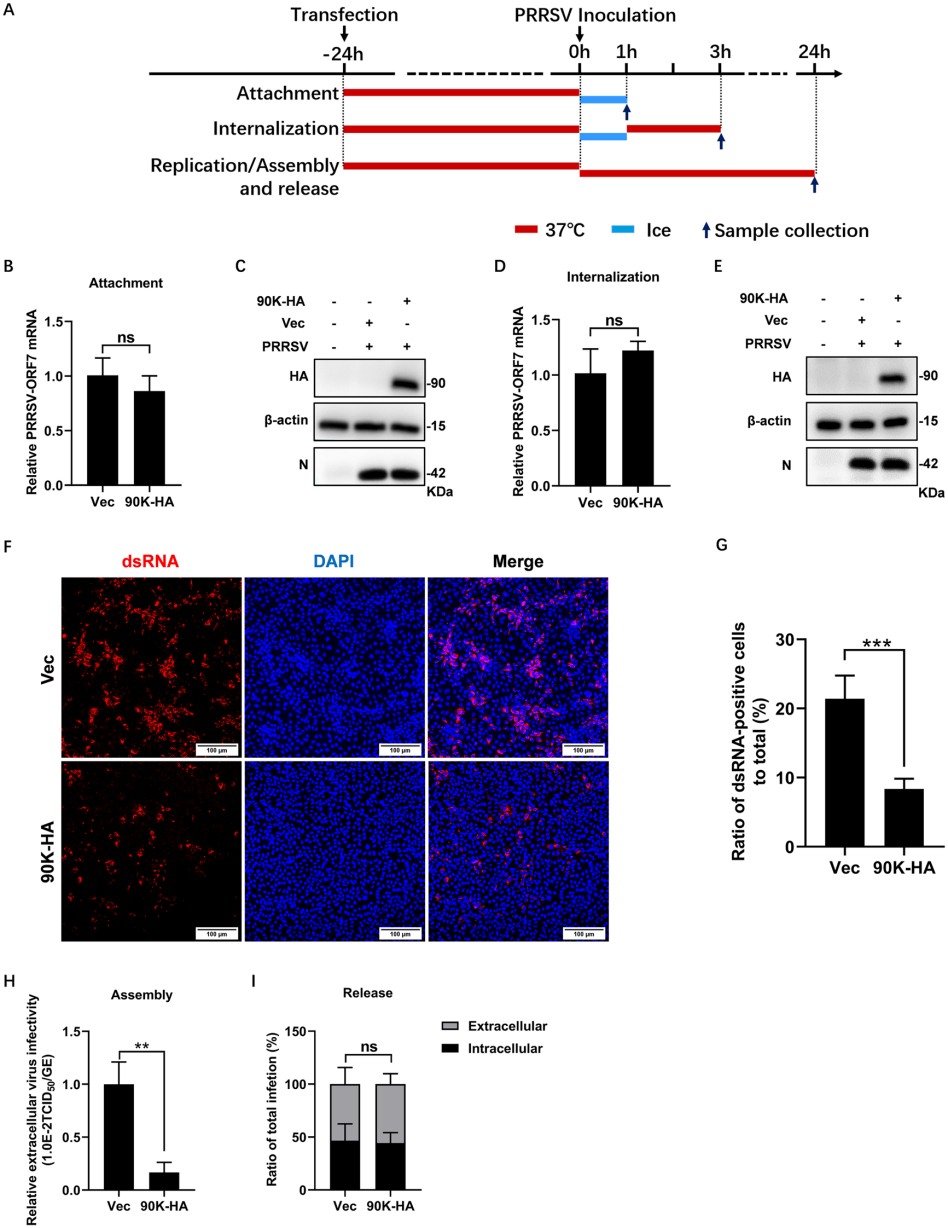
**90K inhibits PRRSV RNA replication and virion assembly.** **A** Overview of the experiment designed for examining PRRSV attachment, internalization, replication, assembly, and release. **B** and **C** MARC-145 cells were transfected with 90K-HA or Vec. At 24 hpt, the cells were inoculated with PRRSV BB0907 (MOI = 10) and kept on ice for 1 h. Cells were then washed three times with ice-cold PBS. The expression levels of PRRSV-N were quantified using qRT-PCR (**B**) and WB (**C**). **D** and **E** Transfected cells were inoculated with PRRSV BB0907 (MOI = 10) and cultured on ice for 1 h, and were then shifted to 37 °C. After 2 h, cells were washed three times with ice-cold citrate buffer. PRRSV-N expression levels were quantified by qRT-PCR (**D**) and WB (**E**). **F** Transfected cells were inoculated with PRRSV BB0907 (MOI = 1). At 24 hpi, cells were fixed and inoculated with anti-dsRNA antibody (red). Nuclei were stained with DAPI (blue). Scale bars, 100 μm. **G** Random fields of view were recorded and the ratio of dsRNA positive cells were quantified with ImageJ. **H** Transfected cells were infected with PRRSV BB0907 (MOI = 1) for 24 h. Viral assembly efficiency was represented as the ratio of TCID_50_/mL to the total number of PRRSV genome equivalents (GE). **I** The efficiency of virus release was determined as the ratio of intra- and extracellular infectivity relative to the total infectivity. The bars represent means ± SD from three independent experiments. (**, *p* < 0.01; ***, *p* < 0.001; ns, not significant).

Next, we explored the effects of 90K on different stages of the PRRSV life cycle (Figure 4A). To determine whether 90K inhibited PRRSV attachment, MARC-145 cells transfected with 90K-HA or Vec were incubated with PRRSV on ice for 1 h. After removing the unbound viruses, qRT-PCR and WB analysis were performed to quantify the bound viruses. As shown in Figures 4B and C, there was no significant difference between the cells transfected with 90K-HA and those transfected with Vec. To determine whether 90K affected PRRSV internalization, transfected cells were incubated with PRRSV on ice for 1 h, followed by a 2 h incubation at 37 °C to facilitate internalization. Uninternalized viruses were removed using ice-cold citrate buffer. The qRT-PCR and WB analysis indicated that 90K had no effect on PRRSV internalization (Figures 4D and E). We next investigated whether 90K affected the replication of PRRSV viral RNA. Transfected MARC-145 cells were infected with PRRSV for 24 h and then double-stranded RNA (dsRNA), the intermediate of viral RNA replication, was detected by IFA. Figures 4F and G demonstrate that overexpression of 90K significantly reduced the levels of PRRSV dsRNA. To further investigate how 90K contributes to PRRSV particle assembly, we analyzed the infectivity of cell supernatants by comparing infectious titers to viral RNA levels. Transfection with 90K-HA resulted in a significant decrease in PRRSV particle assembly (Figure 4H). PRRSV release was represented as the relative amounts of intra- and extracellular infectivity compared to the total infectivity. The results indicate that 90K did not affect viral release levels (Figure 4I). In conclusion, our findings indicate that 90K primarily affects PRRSV viral RNA replication and particle assembly during the viral life cycle.

### 90K interacts with PRRSV-GP2a, GP3 and GP5

**Figure 5:**
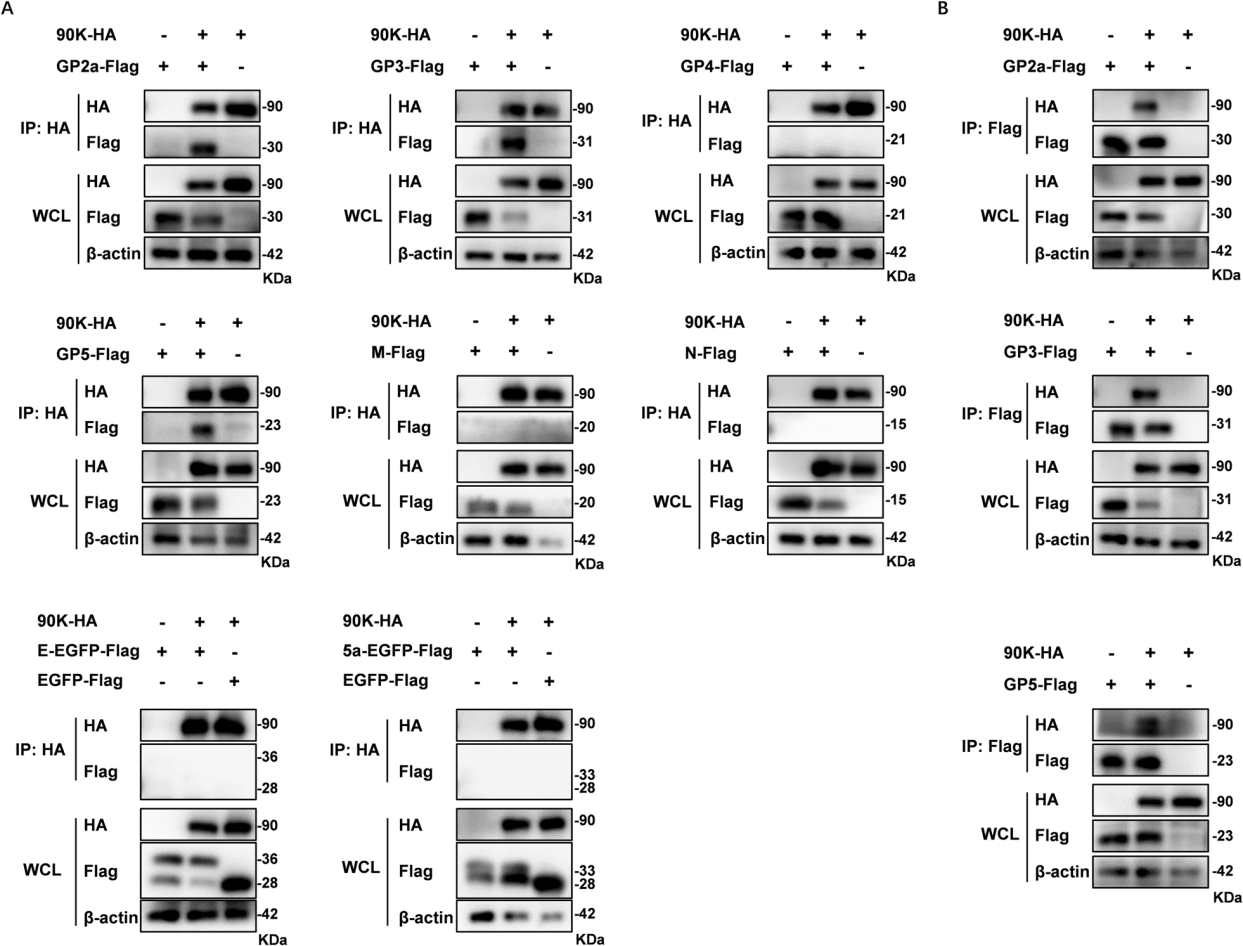
**90K interacts with multiple PRRSV structural proteins.** **A** HEK-293 T cells were co-transfected with plasmids that express HA-tagged 90K and Flag-tagged PRRSV structural proteins. After 48 h, the cell lysates were immunoprecipitated with anti-HA antibodies. Whole-cell lysate (WCL) and immunoprecipitation (IP) complexes were analyzed using immunoblotting with anti-HA and anti-Flag antibodies. **B** HEK-293 T cells were co-transfected with plasmids for Flag-tagged PRRSV structural proteins and HA-tagged 90K. At 48 hpt, cells were lysed and immunoprecipitated with anti-Flag antibodies. WCL and IP complexes were used for immunoblotting analysis with anti-Flag and anti-HA antibodies.

**Figure 6:**
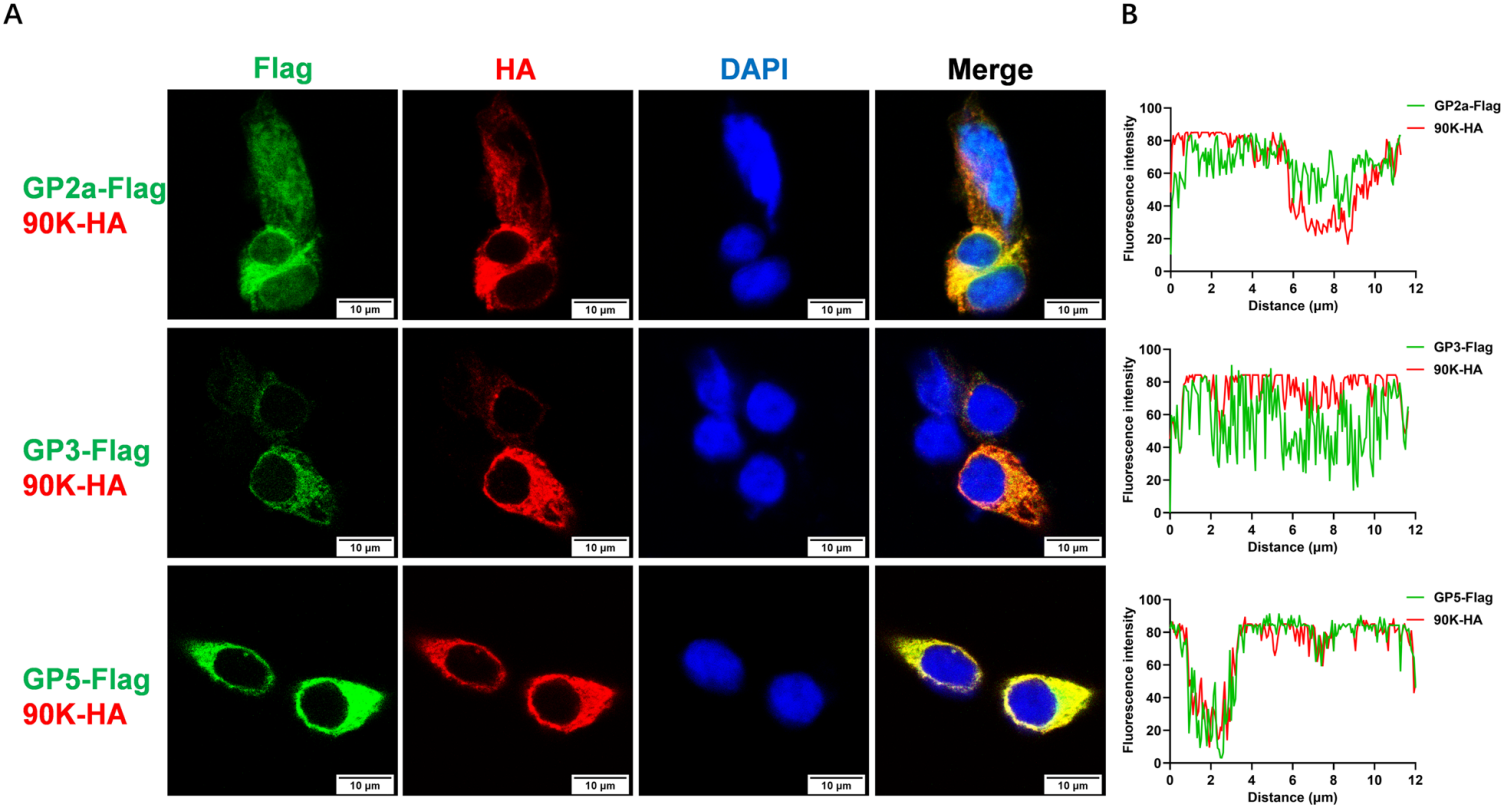
**90K colocalize with PRRSV-GP2a, GP3 and GP5 in the cytoplasm.** **A** HEK-293 T cells were co-transfected with 90K-HA and plasmids encoding Flag-tagged PRRSV-GP2, GP3 or GP5. At 24 hpt, the cells were fixed and analyzed using indirect immunofluorescence to identify Flag-tagged structural proteins (green) and HA-tagged 90K (red). The nuclei were stained with DAPI (blue). Scale bars, 10 μm. **B** The graphs illustrate the use of ImageJ software to conduct line scan analysis, showing the relative positions of the two markers.

A previous study showed that 90K can interact with HIV Gag and inhibit the formation of progeny virions [21]. To determine if 90K interacts with PRRSV structural proteins, we conducted a Co-IP assay. HEK-293T cells were co-transfected with 90K-HA and various vectors that express PRRSV structural proteins (Flag-tagged). To overcome detection limitations imposed by the low molecular weights of PRRSV-E and 5a proteins, Flag-tagged EGFP fusion constructs (E-EGFP-Flag and 5a-EGFP-Flag) were generated and utilized in our experiments. We found 90K could interact with PRRSV-GP2a, GP3 and GP5 (Figure 5A). Reverse Co-IP experiment was also used to confirm these interactions, and 90K could be efficiently coimmunoprecipitated with these structural proteins (Figure 5B). Furthermore, we co-transfected HEK-293 T cells with plasmids encoding 90K and PRRSV-GP2, GP3 or GP5 to investigate their intracellular distribution. As illustrated in Figure 6, 90K efficiently co-localizes with these proteins in the cytoplasm.

### 90K potentiates GP3 degradation through the ubiquitin–proteasome pathway

**Figure 7:**
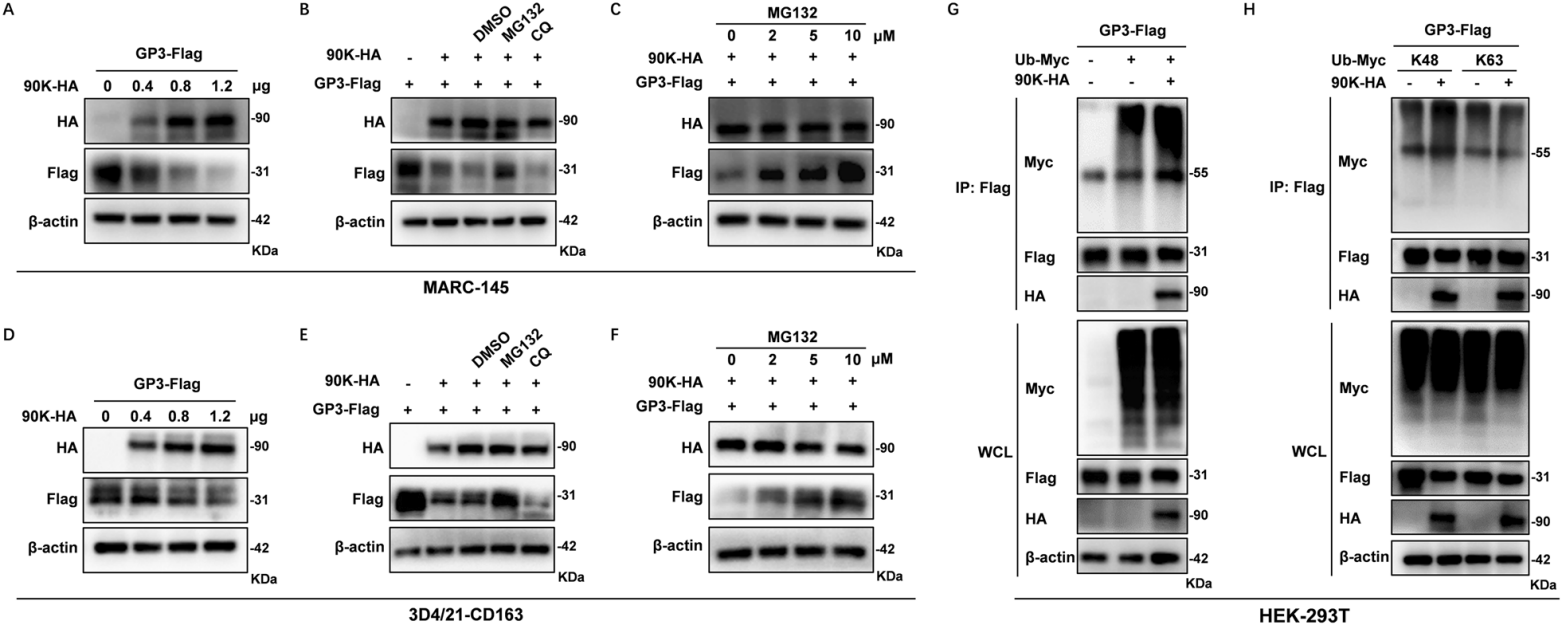
**90K degrades PRRSV-GP3 through a proteasome-dependent pathway.** **A** and **D** MARC-145 (**A**) or 3D4/21-CD163 (**D**) cells were co-transfected with plasmid encoding Flag-tagged GP3 (GP3-Flag) and increasing doses of 90K-HA (0, 0.4, 0.8, or 1.2 μg). The expression levels of PRRSV-GP3 were measured using WB at 24 hpt. **B** and **E** MARC-145 (**B**) or 3D4/21-CD163 (**E**) cells were co-transfected with GP3-Flag and 90K-HA. At 14 hpt, cells were treated with either dimethyl sulfoxide (DMSO), MG132 (10 μM), or CQ (160 μM) for 10 h. GP3-Flag expression levels were detected by WB. **C** and **F** MARC-145 (**C**) or 3D4/21-CD163 (**F**) cells were co-transfected with GP3-Flag and 90K-HA and treated with increasing doses of MG132 (0, 2, 5, or 10 μM). The cell lysates were analyzed by WB using the specified antibodies. **G** HEK-293 T cells were co-transfected with GP3-Flag, Ub-Myc, and 90K-HA or Vec. At 36 hpt, cells were collected for Co-IP assay with an anti-Flag antibody. This was followed by WB analysis with indicated antibodies. **H** HEK-293 T cells were co-transfected with plasmids encoding GP3-HA, 90K-HA, and Myc-tagged Ub mutants (Ub-K48 or Ub-K63). At 24 hpt, Co-IP assay was performed as described above and then analyzed by WB.

Interestingly, the Co-IP results showed that the expression level of PRRSV-GP3 protein was significantly reduced in the 90K-HA overexpression group (Figure 5). To confirm this observation, MARC-145 cells were co-transfected with different amounts of the 90K-HA construct and the PRRSV-GP3 encoding plasmid (GP3-Flag). As shown in Figure 7A, GP3 expression gradually reduced with increased expression of 90K, indicating that 90K induces degradation of PRRSV-GP3.

The ubiquitin–proteasome system and autolysosome pathways are main protein degradation machineries in eukaryotic cells. To investigate how GP3 is degraded, the proteasome inhibitor MG-132 or lysosome inhibitor chloroquine (CQ) was added to cells co-transfected with 90K-HA and GP3 expression plasmids. The results indicated that treatment with MG-132 increased GP3 expression, while CQ had no significant effect (Figure 7B). To further confirm this observation, we conducted a dose-dependent experiment. As shown in Figure 7C, MG-132 could recover GP3 expression in a dose dependent manner. Consistent with findings in MARC-145 cells, overexpression of 90K led to a dose-dependent reduction of PRRSV-GP3 expression in 3D4/21-CD163 cells (Figure 7D). Co-treatment with MG-132 reversed this suppression in a concentration-dependent way, whereas CQ did not counteract the effects of 90K (Figures 7E and F). The consistent results confirm that 90K uses the proteasomal pathway to promote the degradation of GP3, rather than the lysosomal pathway.

Therefore, a Co-IP assay was performed to determine if the ubiquitination level of GP3 could be elevated in cells co-transfected with 90K-HA, GP3-Flag, and the wild-type Ub plasmid (Myc-tagged). The results showed that the ubiquitination level of GP3 was increased by 90K (Figure 7G). These results indicated that 90K promoted GP3 degradation via the ubiquitin–proteasome pathway.

### 90K targets the K217 site of GP3 for K48-linked ubiquitination

The K48-linked polyubiquitin chain primarily mediates proteasomal degradation. Therefore, we analyzed the outcome of K48-linked ubiquitination of PRRSV-GP3. HEK-293 T cells were co-transfected with 90K-HA, GP3-Flag and the plasmid encoding Myc-tagged Ub-K48 (in which all lysine residues of Ub were mutated except K48). As expected, the Co-IP results confirmed that PRRSV-GP3 is modified by the K48-linked Ub chain (Figure 7H). We also excluded K63-linkage of the Ub chain in PRRSV-GP3 in cells co-transfected with 90K-HA, GP3-Flag, and the plasmid encoding Myc-tagged Ub-K63 (in which only the lysine residue of K63 was maintained).

**Figure 8:**
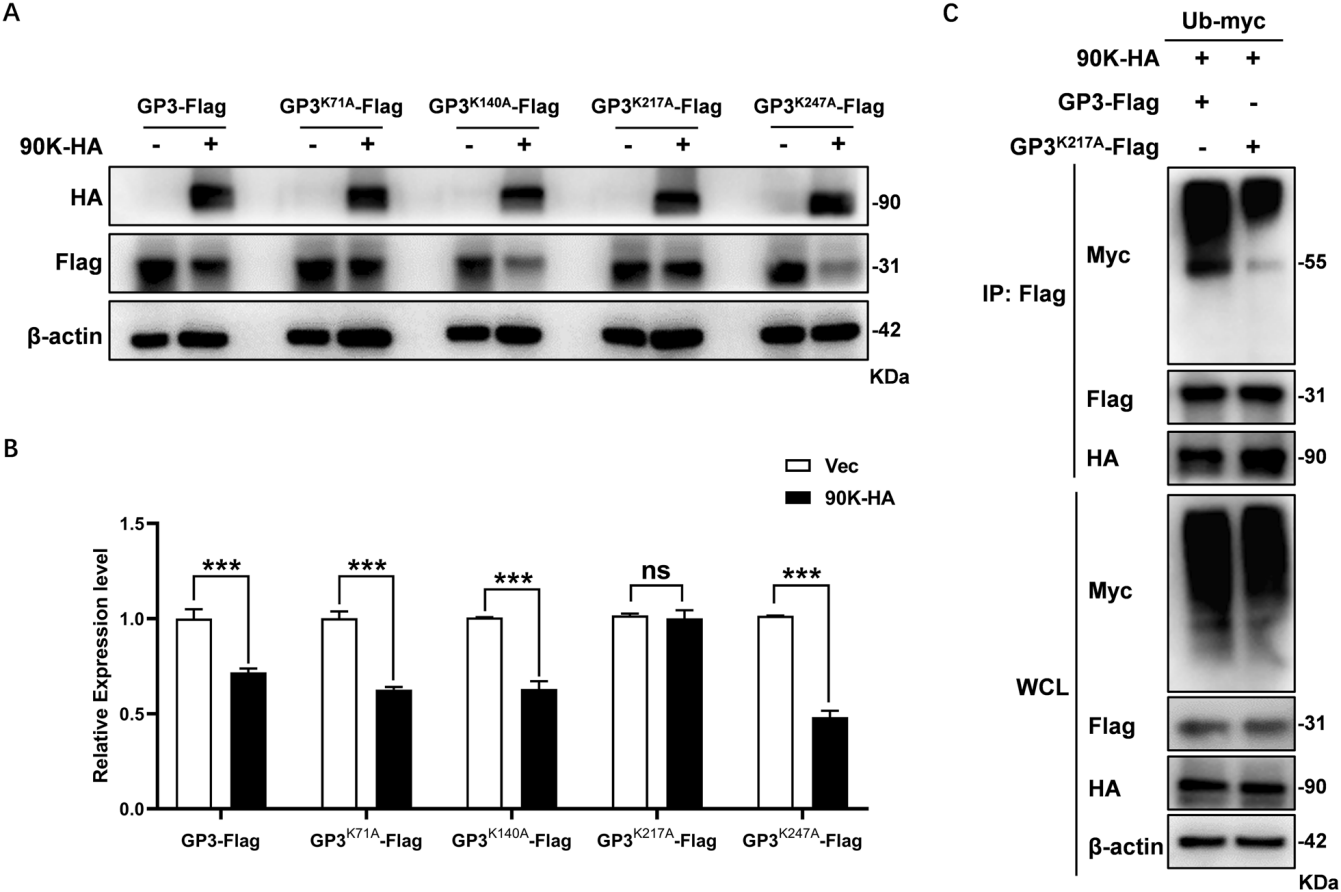
**90K targets the K217 site of GP3 for ubiquitination.** **A** HEK-293 T cells were co-transfected with expression plasmids containing 90K-HA and either GP3-Flag or its mutants (GP3^K71A^, GP3^K140A^, GP3^K217A^, or GP3^K247A^). At 24 hpt, the cells were collected for WB. **B** At least three independent replicates were analyzed by densitometry to determine the relative protein expression levels. The bars represent the means ± SD from three independent experiments. (***, *p* < 0.001; ns, not significant). **C** HEK-293 T cells were co-transfected with plasmids encoding 90K-HA, Ub-Myc and either Flag-tagged GP3 or its mutant GP3^K217A^. At 24 hpt, cells were collected for Co-IP with an anti-Flag antibody. The WCL and IP complexes were analyzed by immunoblotting with indicated antibodies.

To identify which of the four lysine residues (K71, K140, K217, and K247) in GP3 is responsible for linking Ub chain, we created four mutants, each with a lysine-to-alanine (K to A) mutation. MARC-145 cells were co-transfected with the 90K-HA plasmid and the plasmid expressing GP3 variant (GP3^K71A^, GP3^K140A^, GP3^K217A^ or GP3^K247A^). We found that GP3^K217A^ prevented the degradation of GP3 by 90K, indicating that K217 is the site of ubiquitination (Figures 8A and B). Additionally, HEK-293T cells were co-transfected with 90K-HA, Ub-Myc and either GP3-Flag or GP3^K217A^-Flag. Co-IP showed that wild type GP3 was ubiquitinated, while GP3^K217A^ was not (Figure 8C). Together, these results indicate that PRRSV-GP3 undergoes K48-linked polyubiquitination at K217 for proteasomal degradation.

### 90K induces the expression of interferon (IFN) and inflammatory cytokines by activating both NF-κB and IRF3

**Figure 9:**
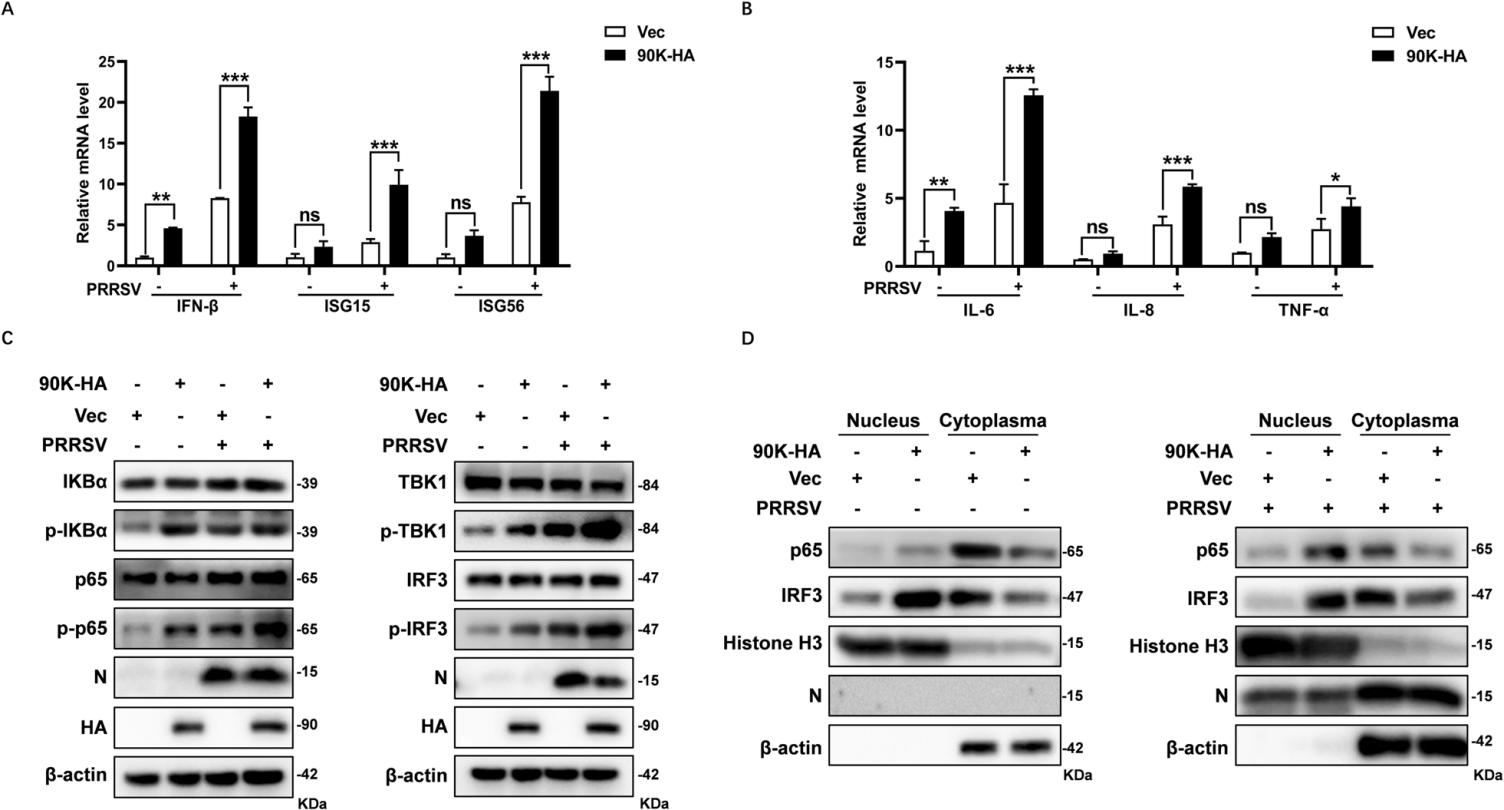
**90K induces cytokine expression by activating NF-κB and IRF3.** MARC-145 cells were co-transfected with either 90K-HA or Vec for 24 h. Afterward, the cells were either infected or not infected with PRRSV (MOI = 1). **A** and **B** At 36 h, cells were collected for qRT-PCR to measure the expression levels of indicated cytokines. ** C** Cell lysates were analyzed by WB to determine the expression levels of indicated signal molecules. **D** Cytosolic and nuclear extracts were prepared and subjected to WB analysis to detect the translocation of NF-κB and IRF3. Histone-H3 and β-actin were used as markers for nuclear and cytosolic fraction, respectively. The bars represent the means ± SD of three independent experiments. (*, *p* < 0.1; **, *p* < 0.01; ***, *p* < 0.001; ns, not significant).

Cytokines can establish antiviral status in cells. Since 90K restricts PRRSV replication, we investigated whether it also regulates the production of IFN and inflammatory cytokines. Using qRT-PCR, we detected increased mRNA levels of IFN-β, IL-6, IL-8, and TNF-α in MARC-145 cells overexpressing 90K and infected with PRRSV (Figures 9A and B). Furthermore, 90K overexpression also increased the expression of ISG15 and ISG56 induced by PRRSV. These findings suggest that 90K enhanced the expression of IFN and inflammatory cytokines expression triggered by PRRSV.

To identify the molecular mechanism, MARC-145 cells were transfected with 90K-HA and then infected with PRRSV. The results showed that overexpression of 90K increased the phosphorylation levels of IκBα, TBK1, p65 (the NF-κB subunit), and IRF3, while the total protein levels of these proteins remained unchanged (Figure 9C). This increase in phosphorylation correlated with enhanced translocation of p65 and IRF3 from the cytosol to the nucleus in PRRSV infected MARC-145 cells transfected with 90K-HA (Figure 9D). Notably, transfecting cells with 90K-HA alone, without PRRSV infection, effectively induced the phosphorylation and nuclear translocation of both p65 and IRF3. This observation indicates that the relevant signaling pathways are directly activated by the overexpression of 90K, rather than being indirectly affected by the suppression of PRRSV replication. Therefore, 90K increases IFN and inflammatory cytokine expression by activating NF-κB and IRF3. This activation triggers antiviral innate immunity and inhibits PRRSV replication.

## Discussion

Currently, commercial vaccines offer limited protection against PRRS, so identifying host factors that resist PRRSV infection could lead to new control strategies. Numerous host proteins can inhibit PRRSV replication. Some of these proteins, including Mx1, Mx2, Viperin, CH25H, bone marrow stromal cell antigen 2 (BST2), and NoNo can be induced by IFNs [7, 12, 16, 17, 30, 39]. 90K is a glycoprotein encoded by the LGALS3BP gene and is also an IFN-stimulated gene (ISG) product [15, 23]. Notably, the expression of 90K and its susceptibility to IFN stimulation vary depending on the cell type. T-cells show very low and undetectable levels of 90K expression and do not respond to IFN-induced increases in 90K protein levels [22]. To investigate if IFNs can induce 90K expression in cells susceptible to PRRSV, we treated MARC-145 cells and primary PAMs with IFN-α. As shown in Additional file 4, IFN-α effectively induced 90K mRNA and protein expression levels in both cell types in a dose-dependent manner. Furthermore, the expression of 90K can also be induced during both chronic and acute viral infections [22]. For example, high concentrations of 90K can be observed in patients infected with HIV, hepatitis B virus (HBV), hepatitis C virus (HCV), dengue virus (DENV), hantavirus, or SARS-CoV-2 [1, 10, 20, 21]. Elevated mRNA levels of 90K are also found in embryonic fibroblasts (MEFs) infected with influenza A virus (IAV), vesicular stomatitis virus (VSV), or herpes simplex virus (HSV) [35]. Functionally, 90K demonstrates potential antiviral roles against infections caused by HIV, HBV, IAV, VSV, and HSV [21, 31, 35]. However, the expression profile of 90K during PRRSV infection and its effect on PRRSV replication are still unclear. Our study revealed that PRRSV infection increased 90K expression at both the transcriptional and translational levels in vitro. Overexpression of 90K reduced PRRSV replication, while knockdown of 90K enhanced viral replication, suggesting that 90K may serve as a limiting host factor for PRRSV infection. We also observed that the upregulation of 90K slightly decreased with prolonged PRRSV infection, particularly in MARC-145 cells. This suggests that PRRSV may have developed a mechanism to reduce 90K expression and counteract its antiviral effects, underscoring the complexity of virus-host interactions. Knowledge of mechanisms about regulation of 90K expression is rare, and the specific mechanism of regulation of 90K during PRRSV replication deserves further investigation. Notably, our study only examined in vitro cellular infection models and did not investigate the expression profiles of 90K in PRRSV-infected piglets in vivo. Furthermore, a review of existing transcriptomic and proteomic studies found no conclusive evidence of increased 90K expression levels in PRRSV-infected piglets [28, 33]. Collectively, the expression dynamics of 90K in PRRSV-infected piglets require further investigation.

Although broad antiviral activity of 90K has been documented, there are few explanations regarding its underlying mechanisms. In the context of HIV infection, 90K disrupts the processing of viral gp160/Env into gp120 and gp41, which reduces the infectivity of progeny virions [21]. Additionally, 90K can form a complex with the viral protein Gag and the cytoskeletal component vimentin, inhibiting the formation of progeny virions [31]. 90K can interact with recombinant adeno-associated viruses (rAAV), including rAAV-1, rAAV-5, and rAAV-6 [6]. Also, 90K co-purifies with hantavirus particles likely via direct interaction with viral GPs [22]. In summary, 90K may associate with viruses to inhibit their infectivity or disrupt the assembly and release of progeny virions. In our study, we examined how 90K influences various stages of the PRRSV life cycle and discovered that it primarily impacts viral genome replication and particle assembly. Co-IP assays were conducted to determine whether 90K interferes with viral assembly by directly interacting with PRRSV structural proteins. Our findings indicate that 90K interacts with several PRRSV envelope proteins, including GP2a, GP3, and GP5. GP2a and GP3 are minor envelope proteins that can form a heterotrimer with GP4 on the virion envelope [34]. GP5 is viral major envelope protein, which forms a disulfide-linked heterodimer with M [5]. Given that all these envelope proteins are crucial for PRRSV assembly, the interaction between 90K and these proteins may impact the assembly of viral particles. Interestingly, our Co-IP assay revealed that 90K overexpression downregulated GP3 expression levels. Previous studies have shown that certain host proteins target PRRSV proteins for degradation. For example, PSMB4 and CH25H degrade PRRSV-nsp1α via the autolysosome and ubiquitin–proteasome pathways, respectively [17, 36]. PSMB1 degrades PRRSV-nsp12 through the autolysosome pathway [19]. In contrast, bone marrow stromal cell antigen 2 (BST2) and RING finger protein 114 (RNF114) facilitate nsp12 degradation via a proteasome-dependent pathway [39]. In this study, we demonstrated that 90K promoted GP3 degradation through the ubiquitin–proteasome pathway. Notably, our study found that the 90K protein does not significantly promote the degradation of GP2a and GP5. The interactions between 90K and these proteins are proposed to influence viral proliferation by potentially hindering the formation of GP2a-GP3-GP4 and GP5-M complexes. Additionally, they might disrupt the trafficking and processing of these proteins from the endoplasmic reticulum to the Golgi apparatus, which could affect virion assembly. In conclusion, the precise mechanisms remain unclear and require further investigation.

Our study also identified site K217 in GP3 as the key ubiquitination site. The conservation profile of this GP3 residue was subsequently analyzed across diverse PRRSV genotypes. Surprisingly, the K217 was conserved only in HP-PRRSV strains (data not shown). Given that 90K also inhibits PRRSV genome replication, we questioned whether it could also activate innate immunity to inhibit viral replication. During PRRSV infection, host-specific pattern-recognition receptors (PRRs), such as Toll-like receptors (TLRs) and RIG-I-like receptors (RLRs), recognize pathogen-associated molecular patterns (PAMPs) and activate the innate immune response [4]. Consequently, transcription factors, including NF-κB and IRF3, become phosphorylated and translocate from the cytoplasm to the nucleus [11, 26]. Following their activation, IFNs and pro-inflammatory cytokines are produced, prompting the transcription of a diverse array of antiviral and inflammatory genes that contribute to the innate antiviral immune response. We studied how 90K affects PRRSV-induced cytokines and discovered that it increases the mRNA levels of IFN-β and pro-inflammatory cytokines. Additionally, 90K also increased the mRNA levels of PRRSV-induced ISGs. This finding explains how 90K can inhibit the classical PRRSV S1 strain and NADC30-like PRRSV FJ1402 strain, which lack the K217 residue in their GP3 proteins. We also investigated how 90K affects the activation of IRF3 and NF-κB, including the upstream events. The results indicated that 90K enhances innate immunity, helping to combat PRRSV infection.

**Figure 10:**
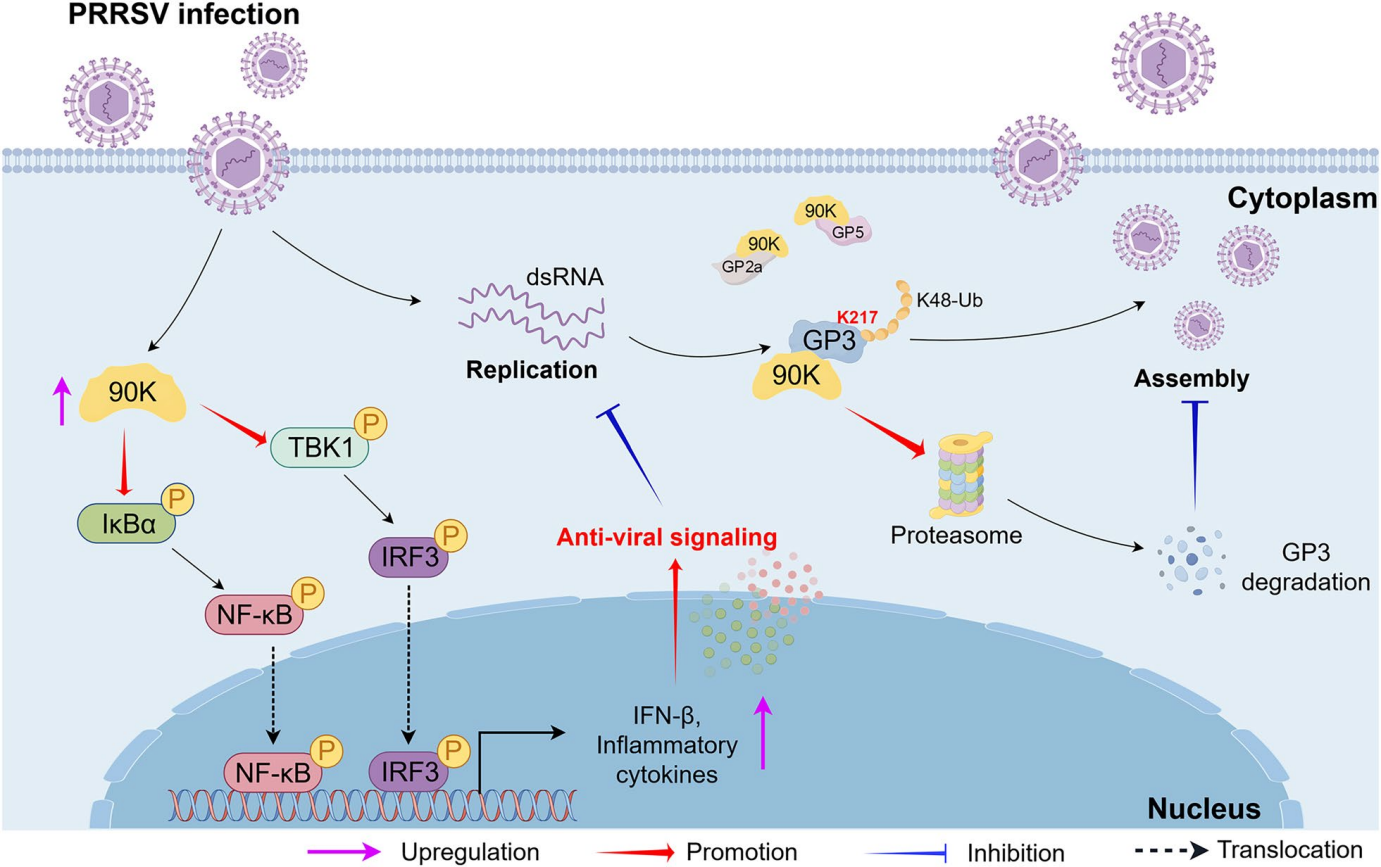
**Model of the mechanism by which 90K suppresses PRRSV replication.** 90K can be induced during PRRSV infection. 90K restricts PRRSV RNA replication and assembly. Mechanistically, 90K interacts with several PRRSV structural proteins, such as GP2a, GP3, and GP5. Additionally, it targets the K217 site of GP3 for K48-linked ubiquitination, which leads to its degradation through a proteasome-dependent pathway. Furthermore, this protein upregulates the expression of p-p65 and p-IRF3, facilitating the translocation of p65 and IRF3 from the cytoplasm to the nucleus, which promotes the production of IFNs and inflammatory cytokines. By Figdraw.

In conclusion, our study reveals a new mechanism for 90K in antiviral replication during PRRSV infection. Specifically, 90K targets K217 in GP3 for K48-linked ubiquitination, promoting the degradation of GP3 through the proteasomal pathway. Moreover, it enhances antiviral immunity by activating NF-κB and IRF3, leading to the upregulation of interferons and inflammatory cytokine levels (Figure 10). However, the precise functional role of 90K in PRRSV-infected piglets remains poorly characterized. Current in vitro evidence suggests a plausible dual antiviral mechanism involving 90K-mediated degradation of viral structural components coupled with potentiation of innate immune signaling pathways, though this hypothesis requires rigorous validation in porcine infection models.

## Supplementary Information

**Additional file 1. Primers used for the construction of PRRSV-GP3 mutants.**

**Additional file 2. The siRNA sequences utilized in this research.**

**Additional file 3. Primers utilized for the qRT-PCR.**

**Additional file 4. The protein 90K can be induced in both MARC-145 cells and PAMs following treatment with IFN-α. **MARC-145 cells or PAMs were treated with IFN-α at concentrations of 0, 50, 100, or 200 ng/mL for 24 h. The mRNA and protein levels of 90K were analyzed using qRT-PCR (A and C) and western blot (B and D). The bars represent the means ± SD from three independent experiments. (***, *p* < 0.001).

## Data Availability

All data generated or analyzed during this study are included in this published article and its supplementary information files.
