# Supplementary material for: LGALS3BP/90K suppresses porcine reproductive and respiratory syndrome virus replication by enhancing GP3 degradation and stimulating innate immunity
Source: Vet Res. 2025 Jun 20;56:121. doi: 10.1186/s13567-025-01556-2 (PMC12180180; doi:10.1186/s13567-025-01556-2)
Supplement: Supplementary file 1 — Additional file 1. Primers used for the construction of PRRSV-GP3 mutants. [file 13567_2025_1556_MOESM1_ESM.docx]

**Additional file 1. Primers used for the construction of PRRSV-GP3 mutants.**

| Primer | Nucleotide Sequence (5'—3') |
| --- | --- |
| GP3-71K/A-F | ACCCTTGAACCCGGCGCGTCTTTTTGGTGCAG |
| GP3-71K/A-R | CTGCACCAAAAAGACGCGCCGGGTTCAAGGGT |
| GP3-140K/A-F | GTTTATGTTGACATCGCGCACCAATTCATCTG |
| GP3-140K/A-R | CAGATGAATTGGTGCGCGATGTCAACATAAAC |
| GP3-217K/A-F | GTCTTTCGGACATTGCAACCAACACCACCGCA |
| GP3-217K/A-R | TGCGGTGGTGTTGGTTGCAATGTCCGAAAGAC |
| GP3-247K/A-F | CTCCGACGATTCGCAGCAGTTCTCAGTGCCGC |
| GP3-247K/A-R | GCGGCACTGAGAACTGCTGCGAATCGTCGGAG |

F and R represent forward and reverse primers, respectively.
