# Supplementary material for: LGALS3BP/90K suppresses porcine reproductive and respiratory syndrome virus replication by enhancing GP3 degradation and stimulating innate immunity
Source: Vet Res. 2025 Jun 20;56:121. doi: 10.1186/s13567-025-01556-2 (PMC12180180; doi:10.1186/s13567-025-01556-2)
Supplement: Supplementary file 2 — Additional file 2. The siRNA sequences utilized in this research. [file 13567_2025_1556_MOESM2_ESM.docx]

**Additional file 2. The siRNA sequences utilized in this research.**

| Primer | Nucleotide Sequence (5'—3') |
| --- | --- |
| si90K | GAAGCUCUGCCUGCAGUUCTT |
| siNC | UUCUCCGAACGUGUCACGUTT |
