# Supplementary material for: LGALS3BP/90K suppresses porcine reproductive and respiratory syndrome virus replication by enhancing GP3 degradation and stimulating innate immunity
Source: Vet Res. 2025 Jun 20;56:121. doi: 10.1186/s13567-025-01556-2 (PMC12180180; doi:10.1186/s13567-025-01556-2)
Supplement: Supplementary file 3 — Additional file 3. Primers utilized for the qRT-PCR. [file 13567_2025_1556_MOESM3_ESM.docx]

**Additional file 3. Primers utilized for the qRT-PCR.**

| Primer | Nucleotide Sequence (5’—3’) |
| --- | --- |
| M-90K-F | CATTCAGAGCTGCTGGAACTA |
| M-90K-R | CGTAGGCAATGCTGTGATCT |
| M-IFN-β-F | TAAGCAGCTGCAGCAGTTCCAGAAG |
| M-IFN-β-R | GTCTCATTCCAGCCAGTGCT |
| M-ISG15-F | CACCGTGTTCATGAATCTGC |
| M-ISG15-R | CTTTATTTCCGGCCCTTGAT |
| M-ISG56-F | CCTCCTTGGGTTCGTCTACA |
| M-ISG56-R | GGCTGATATCTGGGTGCCTA |
| M-IL-6-F | GCTGCAGGCACAGAACCA |
| M-IL-6-R | AAAGCTGCGCAGGATGAGA |
| M-IL-8-F | CTGGCGGTGGCTCTCTTG |
| M-IL-8-R | CCTTGGCAAAACTGCACCTT |
| M-TNF-α-F | TCCTCAGCCTCTTCTCCTTCCT |
| M-TNF-α-R | ACTCCAAAGTGCAGCAGACAGA |
| M-actin-F | CTCCATCATGAAGTGCGACGT |
| M-actin-R | GTGATCTCCTTCTGCATCCTGTC |
| P-90K-F | CGTGATCTGCACCAAAGAAAC |
| P-90K-R | CCCGTTTGGCTCTCAAAGA |
| P-β-actin-F | TGAGAACAGCTGCATCCACTT |
| P-β-actin-R | CGAAGGCAGCTCGGAGTT |
| PRRSV-ORF7-F | AAACCAGTCCAGAGGCAAG |
| PRRSV-ORF7-R | TCAGTCGCAAGAGGGAAAT |

M means monkey, P means pig.
