# Supplementary material for: LGALS3BP/90K suppresses porcine reproductive and respiratory syndrome virus replication by enhancing GP3 degradation and stimulating innate immunity
Source: Vet Res. 2025 Jun 20;56:121. doi: 10.1186/s13567-025-01556-2 (PMC12180180; doi:10.1186/s13567-025-01556-2)
Supplement: Supplementary file 4 — Additional file 4. The protein 90K can be induced in both MARC-145 cells and PAMs following treatment with IFN-α. MARC-145 cells or PAMs were treated with IFN-α at concentrations of 0, 50, 100, or 200 ng/mL for 24 h. The mRNA and protein levels of 90K were analyzed using qRT-PCR (A and C) and western blot (B and D). The bars represent the means ± SD from three independent experiments. (***, p < 0.001). [file 13567_2025_1556_MOESM4_ESM.docx]

**Additional file 4. The protein 90K can be induced in both MARC-145 cells and PAMs following treatment with IFN-α.**

**
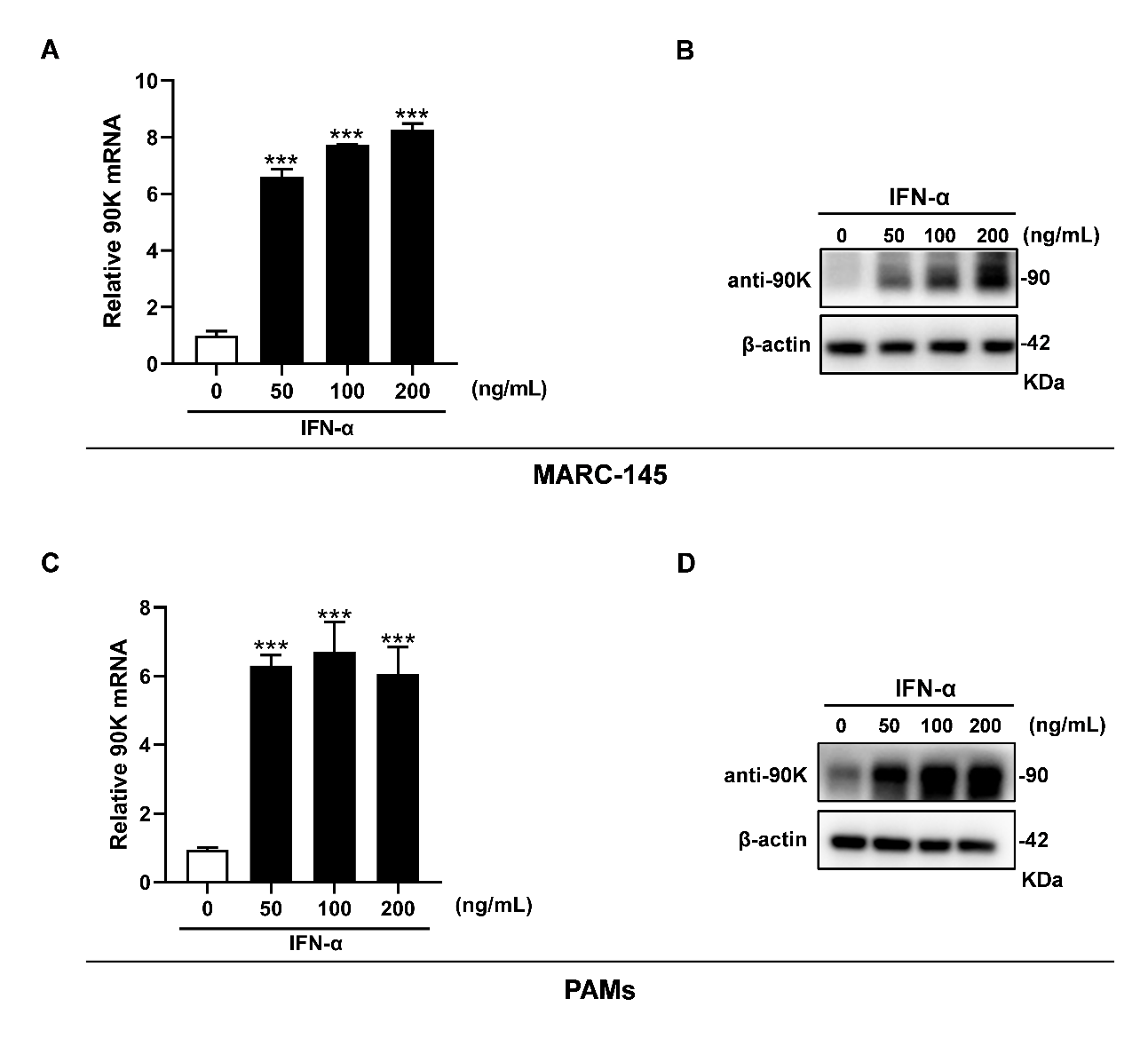
**

MARC-145 cells or PAMs were treated with IFN-α at concentrations of 0, 50, 100, or 200 ng/mL for 24 h. The mRNA and protein levels of 90K were analyzed using qRT-PCR (A and C) and western blot (B and D). The bars represent the means ± SD from three independent experiments. (***, *p* <0.001).
